# Modeling Long-Term Corn Yield Response to Nitrogen Rate and Crop Rotation

**DOI:** 10.3389/fpls.2016.01630

**Published:** 2016-11-11

**Authors:** Laila A. Puntel, John E. Sawyer, Daniel W. Barker, Ranae Dietzel, Hanna Poffenbarger, Michael J. Castellano, Kenneth J. Moore, Peter Thorburn, Sotirios V. Archontoulis

**Affiliations:** ^1^Department of Agronomy, Iowa State University, AmesIA, USA; ^2^Commonwealth Scientific and Industrial Research Organisation Agriculture, St LuciaQLD, Australia

**Keywords:** maize, economic optimum N rate, soybean, soil organic carbon, modeling, APSIM

## Abstract

Improved prediction of optimal N fertilizer rates for corn (*Zea mays L.*) can reduce N losses and increase profits. We tested the ability of the Agricultural Production Systems sIMulator (APSIM) to simulate corn and soybean (*Glycine max L.*) yields, the economic optimum N rate (EONR) using a 16-year field-experiment dataset from central Iowa, USA that included two crop sequences (continuous corn and soybean-corn) and five N fertilizer rates (0, 67, 134, 201, and 268 kg N ha^-1^) applied to corn. Our objectives were to: (a) quantify model prediction accuracy before and after calibration, and report calibration steps; (b) compare crop model-based techniques in estimating optimal N rate for corn; and (c) utilize the calibrated model to explain factors causing year to year variability in yield and optimal N. Results indicated that the model simulated well long-term crop yields response to N (relative root mean square error, RRMSE of 19.6% before and 12.3% after calibration), which provided strong evidence that important soil and crop processes were accounted for in the model. The prediction of EONR was more complex and had greater uncertainty than the prediction of crop yield (RRMSE of 44.5% before and 36.6% after calibration). For long-term site mean EONR predictions, both calibrated and uncalibrated versions can be used as the 16-year mean differences in EONR’s were within the historical N rate error range (40–50 kg N ha^-1^). However, for accurate year-by-year simulation of EONR the calibrated version should be used. Model analysis revealed that higher EONR values in years with above normal spring precipitation were caused by an exponential increase in N loss (denitrification and leaching) with precipitation. We concluded that long-term experimental data were valuable in testing and refining APSIM predictions. The model can be used as a tool to assist N management guidelines in the US Midwest and we identified five avenues on how the model can add value toward agronomic, economic, and environmental sustainability.

## Introduction

The economic optimum nitrogen (N) rate (EONR) is the fertilizer rate at which crop yield increase is not large enough to pay for additional N application, and therefore more N would only result in unnecessary costs (Sawyer et al., 2006). Optimal N input needs to be considered when making N recommendations since it has the potential to improve N use efficiency, crop yield, and profitability as well as to reduce environmental impacts (Wang et al., 2003; Lawlor et al., 2008; Kyveryga et al., 2009; Basso et al., 2016). Nitrogen losses by leaching are proportional to the N rate applied and tend to increase rapidly at rates greater than optimal for crop use (Haghiri et al., 1978; Cooper and Cooke, 1984; Andraski et al., 2000; Randall et al., 2000).

There is tremendous uncertainty and risk associated with prediction of the EONR in corn–based systems, both at the field and sub-field scale (Paz et al., 1999; Scharf et al., 2005; Tremblay et al., 2012). Farmers may attempt to protect corn yield potential with high fertilizer N inputs, which leads to decreased profitability (Lambert et al., 2006) and increased likelihood of environmental contamination (Andraski et al., 2000; Jaynes et al., 2001; Robertson and Groffman, 2007).

A number of approaches have been developed to predict optimal N application rates. These include yield goal-based N recommendations and N budgets (Stanford, 1973, 1982; Stanford and Legg, 1984), pre-plant and pre-sidedress soil nitrate test (PPNT and PSNT, Bundy and Andraski, 1995; Shapiro et al., 2008), Illinois soil nitrogen test (ISNT, Mulvaney et al., 2001), crop canopy sensing (NDVI, Schmidt et al., 2009 and chlorophyll meter, Blackmer and Schepers, 1995; Varvel et al., 1997), and economic maximum return to N (MRTN, Sawyer et al., 2006). Some of these tools are static in that they give the same recommendation regardless of yearly weather or crop/fertilizer prices, or evaluate N status after grain crop harvest. Soil tests or hand-held crop meters are often time consuming, expensive, and/or require periodic and intense sampling (Blackmer et al., 1997; Ma and Dwyer, 1999; Grove and Schwab, 2006; van Es et al., 2007; Lemaire et al., 2008; Franzen et al., 2016). Most current and widely adopted N management practices also assume field-uniformity, recommending N applications that ignore variation in landscape factors such as topography, soil texture, and organic matter (Cassman et al., 2002; Mamo et al., 2003; Scharf et al., 2005), as well as interactions with plant population and hybrid (Ciampitti and Vyn, 2012). Use of precision agriculture technologies (real-time remote sensing, unmanned aerial images, soil mapping, etc.) combined with variable N application have the potential to increase N use efficiency by matching the N requirements within field zones (Dobermann and Cassman, 2002; Ferguson et al., 2002; Mamo et al., 2003; Mulla, 2013). However, the selection of a site-specific optimum N rate is difficult to predict based on the large temporal and spatial variability of the N supply and demand (van Es et al., 2007; Setiyono et al., 2011). Unfortunately, the above approaches have not fully resolved needed improvements from N management and gains in N use efficiency (Raun and Johnson, 1999; Fageria and Baligar, 2005) since N losses from corn-based systems are still high with negative environment impacts (Jaynes et al., 2001; Mitsch et al., 2001).

The challenge in managing N and estimating the optimum N fertilization rate comes from the complex interactions that exist in the dynamic soil-plant-atmosphere system and uncertainty in weather (Havlin et al., 2005; Tremblay and Belec, 2006; Brady and Weil, 2008). Soil N mineralization from SOC and crop N uptake, and N losses are three important components defining the optimum N rate, however, these processes are dynamic and difficult to predict (Cassman et al., 2002). Therefore N management tools that simultaneously consider dynamics in soil organic carbon mineralization, crop growth, weather conditions, and agronomic practices may greatly improve site- and year-specific EONR estimates (Basso et al., 2012, 2016; Dumont et al., 2016). Dynamic cropping system simulation models such as Agricultural Production Systems sIMulator (APSIM; Holzworth et al., 2014), DSSAT (Jones et al., 2003), RZWQM (Ahuja et al., 2000), CropSyst (Stockle et al., 2003), SALUS (Basso et al., 2006), and others have been used to investigate soil-crop-weather dynamics, however, model use has been limited to address long-term optimum N rates (Ma et al., 2007; Basso et al., 2010). The scientific literature is also rich with examples of model applications to improve our understanding of N dynamics and to answer questions that cannot be addressed with field research due to time and cost constraints (Batchelor et al., 2002; Schnebelen et al., 2004; Fountas et al., 2006; Malone et al., 2010; Basso et al., 2012, 2016; Anapalli et al., 2014). However, use of models in practical applications to assist real-life challenges such as N rate guidance is limited because models typically require: (a) a large number of input parameters, which are usually not available (Wallach, 2006; Basso et al., 2012); (b) particular skills to develop model specific input parameters and cultivar coefficients from internet databases; and (c) intensive training for use.

Over the last few years web-applications have been developed to simplify the use of models (e.g., Yield Prophet, Carberry et al., 2009). Furthermore, digital soil and weather databases such as web soil survey^1^ (Soil Survey Staff, 2006) and daymet (Daymet, 1980–2008; Thornton et al., 2012) provide free access to high-resolution input parameters. As a result, the potential of using simulation models to assist with real-life practical problems and especially to predict the risk associated with selecting specific N fertilizer rates has received strong industrial interest (Thorp et al., 2007; Gowda et al., 2008; Nangia et al., 2008). The next challenge to applying models across different scales (within fields, regions, and cropping systems) is to determine prediction accuracy; e.g., how well cropping system models can predict crop yield, N dynamics, and EONR. And if they can predict corn response to N rate, how can this information be used to develop better N rate guidelines.

In this study we used a 16-year field research dataset from a site in central Iowa, USA that included five N rates and two crop sequences to test the ability of the APSIM model (Holzworth et al., 2014) to predict crop yields and optimal N rate for corn. Our specific objectives were to: (a) quantify model prediction accuracy before and after calibration, and report calibration steps; (b) compare crop model-based techniques in estimating optimal N rate for corn; and (c) utilize the calibrated model to explain factors causing year to year variability in yield and optimal N. The APSIM model was selected for use in this study because of its flexibility and easy use in specifying crop rotations via the user interface, capability in simulating long-term dynamics in both soil and crop processes, advanced flexibility in simulating the effect of shallow water table dynamics that are important in this geographic region (Helmers et al., 2012) and previously determined good performance in this geographic region (Malone et al., 2007; Hammer et al., 2009; Lobell et al., 2013; Archontoulis et al., 2014a,b, 2016; Basche et al., 2016; Dietzel et al., 2016; Martinez-Feria et al., 2016).

## Materials and Methods

### Site, Weather, and Experimental Datasets

The field-experiment was conducted at the Agricultural Engineering and Agronomy Research Farm near Ames, Iowa, USA (42° 0′37.50″N, 93°47′22.98″W) on a Clarion loam soil (fine-loamy, mixed, superactive, mesic Typic Hapludoll). The experiment was initiated in 1999 and continuous to the present. For this study we used data from 1999 to 2014 (16-years). The climate at the site is humid continental (warm, rainy summers) with annual precipitation of 900 mm and a mean temperature of 9°C (**Supplementary Figure S1**). Over the 16-year experimental period, crops experienced warm and wet conditions (3 years), cool and wet conditions (3 years), warm and dry conditions (5 years), and cool and dry conditions (5 years; **Supplementary Figure S1**). Years 2008, 2010, and 2014 were the wettest and years 2000, 2011, 2012, and 2013 the driest. Mean annual air temperatures were 16 and 23°C for spring and summer, respectively. Year 2012 was the warmest and year 2008 the coolest (**Supplementary Figure S1**).

The long-term experiment was designed to study the effect of five N fertilizer rates (0, 67, 134, 201, and 268 kg N ha^-1^; hereafter N0, N67, N134, N201, and N268, respectively) on corn yield in continuous corn (CC) and soybean-corn rotation (SC). The experimental design was a randomized complete block design with four replications. Nitrogen fertilizer was applied near planting (± 10–15 days). Specific information on the fertilizer type and application dates are provided in **Supplementary Table S1**. Within the SC rotation, corn and soybean phases were present each year in the rotation: thus a simulation starting with corn in year one and another simulation starting with soybeans on year one were set up. Hereafter SC when the rotation starts with corn in year one (odd numbered years) and a validation set (SC_val) when the rotation starts with soybean in year 1 (even numbered years). Each treatment had four replications. Nitrogen fertilizer was only applied to corn. **Supplementary Table S1** provides management information by year and rotation. Measurements included corn and soybean grain yields each year (expressed at 15.5 and 13% moisture content, respectively). Soil organic carbon measurements were available at 0–15 cm in 1999, 2009, and 2014, and at 0–30 cm in 2009 for CC (Brown et al., 2014; Poffenbarger et al., unpublished).

### The APSIM Modeling Platform

The APSIM (Keating et al., 2003; Holzworth et al., 2014) is an open-source advanced simulator of agricultural systems that combines several process-based models in a modular design. APSIM is a field-scale model that operates mainly on a daily time step. Details about APSIM and its performance across a range of studies can be found at http://www.apsim.info.

### APSIM Configuration and Calibration

Two rounds of APSIM model evaluations were performed; a blind phase (uncalibrated model) where management and cultivar information were used, and a calibrated phase (calibrated model) where crop yield and SOC data were provided into the model. Similar protocols have been used in the AgMIP project (Agricultural Model Inter-Comparison and Improvement Project; Rosenzweig et al., 2013).

#### Blind-Phase Model Parameters and Set-Up

For the blind phase, we first incorporated available management information into APSIM (**Supplementary Table S1**). When required management information was unavailable, we used typical values from the literature relevant to the research site (Abendroth et al., 2011; Pedersen and Licht, 2014). The following input parameters were held constant across the 16-years: planting depth of 5 cm for both crops, plant populations of 8 and 38 plants m^-2^ for corn and soybean, respectively, and November 10th and April 10th dates for fall and spring tillage operations; and corn hybrid (106-day) and soybean variety (2.5 maturity group) values derived from previous studies in the region (Archontoulis et al., 2014a,b; **Supplementary Table S2**). Daily weather data were obtained from the Iowa Environmental Mesonet (2014). Soil profile information was taken from Web Soil Survey (Soil Survey Staff, 2006) and soil-root related parameters were developed following the methodology described in Archontoulis et al. (2014a). The maximum rooting depths for corn and soybean were set to 1.5 and 1.2 m, respectively.

We set up APSIM by connecting the following models: corn and soybean crop models (Keating et al., 2003), Soil N (soil N and C cycling model with default soil temperature model; Probert et al., 1998), SoilWat (a tipping bucket soil water model; Probert et al., 1998); SURFACEOM (residue model; Probert et al., 1998; Thorburn et al., 2001, 2005), and the following management rules: planting, harvesting, fertilizer, tillage, and rotations (Keating et al., 2003). In addition we implemented within the MANAGER module an N deposition rule that simulates atmospheric N deposition as a function of daily precipitation (N deposition in kg N ha^-1^ d^-1^ = 0.01^∗^ precipitation in mm; Holland et al., 2005). On average this added about 7 kg N ha^-1^ year^-1^ into the system. Initial model conditions such as root mass, surface residue mass, soil water, soil nitrate, and SOC pool partitioning were obtained by starting the model 6 years prior to the start of the experiment (**Supplementary Table S3**). Experience using APSIM in this geographic region for simulating corn-soybean production systems has indicated that the fast microbial SOC pool (BIOM) of APSIM requires at least 4 years to stabilize (Basche et al., 2016; Dietzel et al., 2016; Martinez-Feria et al., 2016). Having this pool stabilized is important to remove confounding effects of microbial SOC buildup or decline which affects N dynamics. The APSIM version 7.6 was used on a daily time step. The simulation process was consecutive to account for carry-over effects from year to year, such as soil inorganic nitrogen, soil moisture, root and residue carbon and nitrogen inputs from previous crops.

#### Model Calibration and Testing

In the calibration and testing phase, we used end-of-season grain yields and SOC data to improve predictions. The long-term (end-of-season) data are powerful in detecting weakness in the model (i.e., years with low prediction accuracy), but do not provide guidance on which of the model’s processes or parameters needed to be improved. Therefore, to inform the calibration process, additional information was used: knowledge gained from other APSIM calibration studies in Iowa (Archontoulis et al., 2014a, 2016; Basche et al., 2016; Dietzel et al., 2016), sensitivity techniques, and model behavior analysis coupled with expert judgment (**Supplementary Figure S2**). The odd-numbered years (for CC and SC) were used for calibration and even numbered years for validation (SC_val dataset).

During calibration the following changes in APSIM were made: first, we replaced the default APSIM soil temperature model that uses EPIC model equations (Williams et al., 1984) with a more mechanistic soil temperature model (Campbell, 1985) available in APSIM (soiltemp2). The reason was twofold: (a) soiltemp2 has been found to perform better in Iowa (Archontoulis et al., 2014a; Basche et al., 2016; Dietzel et al., 2016); and (b) soiltemp2 better represents reality than the default model as it accounts for soil temperature changes due to tillage, residue cover, and management practices. Second, we replaced SoilWat with the SWIM soil water model (Huth et al., 2012) available in APSIM. This model allowed simulation of fluctuating shallow groundwater tables, which in this region varies from about 80 to 200 cm (Groundwater, USGS, Iowa Water Science Center). Third, we improved the simulation of soybean residue C:N ratio at harvest because the simulated C:N ratio was low when compared to published data (Johnson et al., 2007) and caused an over-prediction of corn yields in the SC rotation with no N applied. We improved soybean C:N ratio by decreasing the critical N concentration of different plant tissues at physiological maturity by about 20% (**Supplementary Table S2**). Additionally, we decreased the potential N fixation rate (**Supplementary Table S2**) to better match seasonal N fixation estimates to those observed in the literature for this region (Salvagiotti et al., 2008). No changes were made in the corn crop model, although various options were explored via sensitivity analysis. Given all these changes we re-initialized conditions at the start of the simulation on year 1999 (**Supplementary Table S3**).

### Data Analysis

#### Estimation of the Annual Economic Optimum Nitrogen Rate

The relationship between observed or simulated yield and N rate was fit using the quadratic

$$y=a+bx+{cx}^{2}$$

the quadratic-plus-plateau,

$$y=a+bx+{cx}^{2},x<x_{0}$$

$$y=a+bx_{0}+{cx}_{0}^{2},x\geq x_{0}$$

In these equations, *y* represents corn yield (either observed or simulated), *x* is the fertilizer N rate, *a* is the intercept, *b* is the linear coefficient, *c* is the quadratic coefficient, and *x_0_* is the N rate at the join point. The PROC NLIN procedure in SAS (Version 9.4, SAS, 2013). Equations were deemed significant at *p* < 0.05 and the equations with the smallest sums of squares and largest *R*^2^ were selected.

Corn EONR and the yield at the EONR (YEONR) were calculated from the N response equations by setting the first derivative of the fitted response curve equal to the historical price ratio of 5.6:1 N:corn grain price (US$ kg^-1^ N:US$kg^-1^ grain) ratio (Cerrato and Blackmer, 1990; Bullock and Bullock, 1994). The impact of the N:corn grain price ratio on EONR has been well documented in the literature (Cerrato and Blackmer, 1990; Sawyer et al., 2006). In this study, we used a fixed ratio across years similarly to other modeling studies (Basso et al., 2012). Using this approach, we calculated EONR and YEONR values for: (a) the observed data (EONR-Obs, YEONR-Obs); (b) the simulated data from the uncalibrated model (EONR-APSIM-Unc, YEONR-APSIM-Unc); and (c) the simulated data from the calibrated model (EONR-APSIM-Cal, YEONR-Cal).

Additionally, a different technique to calculate an optimal N rate was used (Basso et al., 2016). The calibrated APSIM model was ran for every 5 kg N ha^-1^ increments from 0 to 350 kg N ha^-1^ to simulate corn yields. Then the N rate at which the economic return on N was maximized [hereafter RTN (return to N approach)-APSIM] was estimated by difference: simulated yield times corn price minus fertilizer rate times N cost between two levels of N rate. A value of zero (or near zero) corresponds to the optimum N rate. Same prices for corn grain and N fertilizer was used as with the EONR technique.

The RTN-APSIM technique differs from the EONR-APSIM-Cal in the following way: EONR-APSIM-Cal estimates the economic optimum N rate through regression equations (Eq. 1–3) fitted to five simulated corn yields at 0, 68, 134, 200, and 268 kg N ha^-1^. The RTN-APSIM uses the ability of APSIM to run on any desired N rate increment to predict corn yield (every 5 kg N, from 0 to 350 kg N ha^-1^) and therefore the economic optimum N rate can be identified without use of regression equations. The RTN approach follows a similar methodology to that is currently used for corn N rate recommendations in the USA Midwest (known as the MRTN approach; Sawyer et al., 2006). The difference between RTN-APSIM and MRTN is that the regression equations for MRTN are within a database with extensive N rate response trials and associated regression equations, while RTN-APSIM generates a synthetic database, which depends on the accuracy of the model to predict yields and N response.

#### Estimation of Site Mean Economic Optimum Nitrogen Rate

Two methods were used to estimate the site mean EONR and YEONR: (a) we first averaged individual annual estimates of EONR and YEONR for each rotation, and then calculated the associated standard deviation (SD; across years mean); and (b) we averaged corn yields across years for each rotation and then we estimated EONR and YEONR using regression equation fitting and EONR calculation, which is an approach used for N recommendations (pooled mean, Sawyer et al., 2006). The same methods were used for RTN-APSIM, except no regression equation fitting was required.

#### Statistical Evaluation of Model Performance

To evaluate APSIM model goodness of fit, we used graphical and statistical methods. For the statistical evaluation, we computed the root mean square error (RMSE),

$$RMSE=\sqrt{\frac{\Sigma_{i=1}^{n}{\left(S_{i}-O_{i}\right)}^{2}}{n}}$$

and RRMSE,

$$RRMSE=\frac{RMSE}{\overset{¯}{O}}\times 100$$

where Ō is the mean observed value, *S_i_* is the model estimated value, *O_i_* is the observed value, and n is the number of data pairs. The RMSE summarizes the average difference between observed and predicted values, while RRMSE provides the relative difference. In both cases, the lower the value of the index the better the model performance. In this study, we considered RRMSE ≤ 15% as “good” agreement; 15–30% as “moderate” agreement; and ≥ 30% as “poor” agreement (Liu et al., 2013; Yang et al., 2014).

#### Factors Affecting Optimal Nitrogen Rate Inter-Annual Variability

Regression analysis was performed to identify statistical significant relationships between simulated EONR and explanatory factors. We considered three explanatory factors: yield at optimum N rate, time of N application rate relative to corn planting date, and precipitation sums over different time periods. We used *R*^2^ to evaluate predictability of optimum N rate based on the factors mentioned above.

## Results

### Observed Corn and Soybean Yield Response to N Fertilizer, and Crop Rotation

**FIGURE 1:**
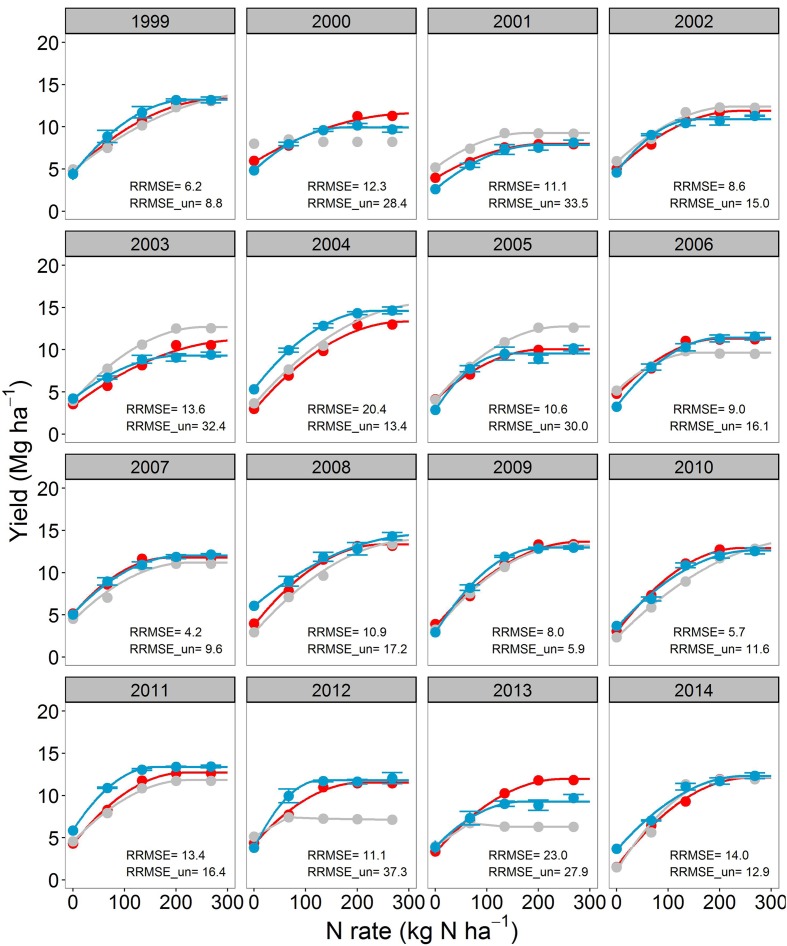
**Corn yield response to N fertilizer for the continuous corn (CC) cropping system.** The blue points with standard errors (*n* = 4) indicate the observations. The gray and red points are Agricultural Production Systems sIMulator (APSIM) simulations before and after calibration, respectively. Continuous lines are regression fits from Eqs. 1–3. When lines are not shown it means that Eqs. 1–3 did not converge. Relative root mean square error for both calibrated (RRMSE) and uncalibrated model (RRMSE_un) are shown for each year.

**FIGURE 2:**
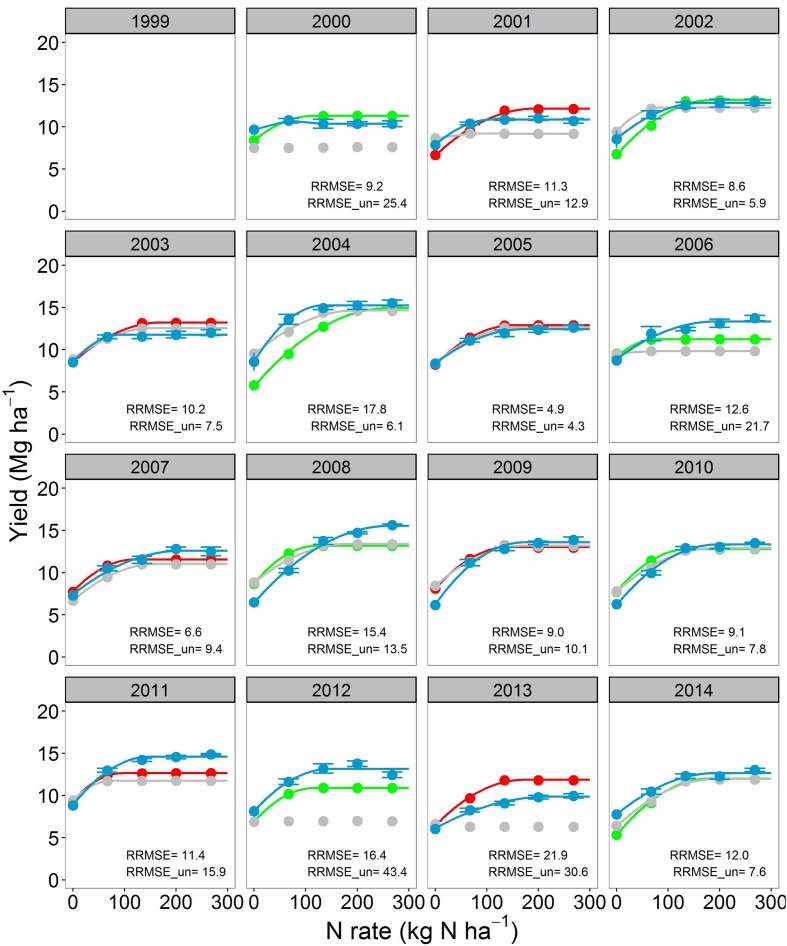
**Corn yield response to N fertilizer for the soybean-corn (SC) cropping system.** The blue points with standard errors (*n* = 4) indicate the observations. The gray, red, and green points indicate uncalibrated, calibrated and validated simulations from the Agricultural Production Systems sIMulator (APSIM) model. Continuous lines are regression fits from Eqs. 1–3. When lines are not shown it means that Eqs. 1–3 did not converge. Relative root mean square error for both calibrated (RRMSE) and uncalibrated model (RRMSE_un) are shown for each year.

**FIGURE 3:**
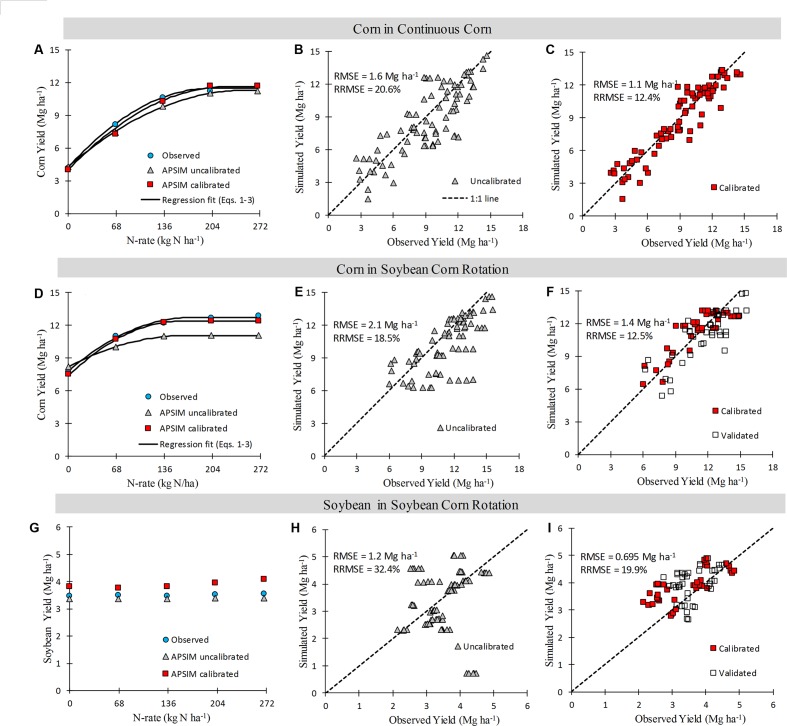
**Sixteen year mean crop yield response to N fertilizer rate **(A, D, and G panels)**,** and observed versus simulated crop yields across years and N rate **(B, C, E, F, H, and I)**. Points are observations or simulations, continuous lines are regression fits from Eqs. 1–3, and broken lines show 1:1 relationship.

**FIGURE 4:**
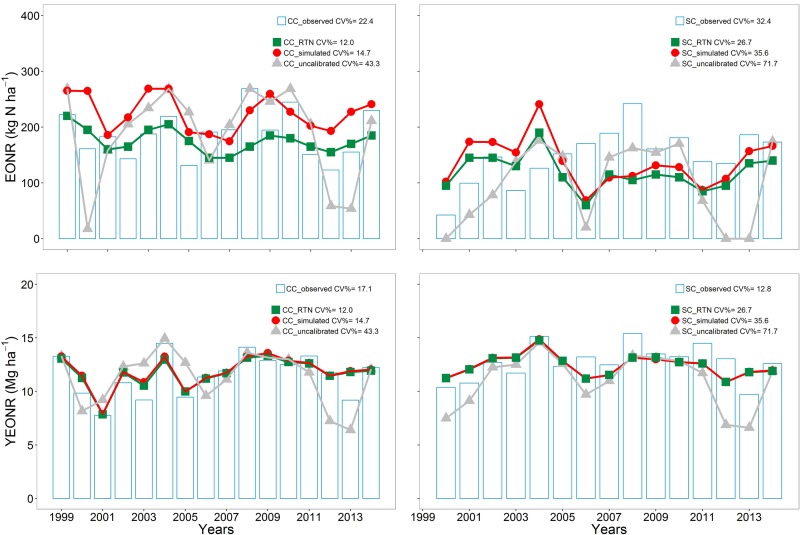
**Economic optimum N rate (EONR) and corn yield at the EONR (YEONR) for every year in CC and SC.** The EONR and YEONR estimates from observations using Eqs. 1-3 are shown as bars. Different color symbols show Agricultural Production Systems sIMulator (APSIM) model simulations: red points calibrated model, gray points uncalibrated model, and green points return to N approach (RTN) from the calibrated model.

Observed corn yield varied across years, N rates, and crop sequences (**Figures 1** and **2**). Yearly variability CC, corn yield averages across years ranged from 4.2 (N0) to 11.6 (N268) Mg ha^-1^ with a maximum yield response to N (difference between N0 and N268 treatment) of 7.6 Mg ha^-1^ (**Figure 3**). In SC, corn yield averages were greater for all N treatments compared to CC, and varied from 7.8 (N0) to 12.9 (N268) Mg ha^-1^ with a maximum yield response to N of 5.1 Mg ha^-1^. At N0, for individual years the largest yield difference between CC and SC was 3.6 Mg ha^-1^. Greater yearly variability in corn yield was observed in CC (coefficient of variation, CV = 17.8%) than in SC (CV = 12.9%). The CV decreased with increasing N rate in CC (from 24.8 to 15.4%), but was consistent across N rate in SC. Across rotations, high corn yields under non-limited N condition were obtained in wet years (precipitation above 1100 mm, e.g., 2008 and 2010; **Figures 1–3**) and low corn yields in dry years (precipitation below 600 mm precipitation, e.g., 2000, 2012, and 2013). Observed soybean yields varied from 2.1 to 4.8 Mg ha^-1^ across years and N rates (**Figure 4**; **Supplementary Figure S2**). The yearly variability in soybean yield had a CV of 19.5%. Soybean yields were not affected by N rates applied to corn (**Figure 3**; **Supplementary Figure S2**).

### Model Accuracy before and after Calibration

#### Simulation of Corn Yields

Overall, across years, N rates and crop sequences, APSIM explained from 50–69% (before calibration) to 67–88% (after calibration) of the observed variability in corn yield (**Figure 3**). The model agreement improved during calibration from moderate (RRMSE = 19.6%, uncalibrated) to good (RRMSE = 12.3%, calibrated) for corn yield prediction (**Figure 3**). In CC, the uncalibrated model simulated corn yield response to N well in 7 years (RRMSE < 15%), moderately well in 6 years (RRMSE 15–30%), and poorly in 3 years (RRMSE > 30%); while after calibration the model simulated yields well in 14 years and moderately well in 3 years (**Figure 1**). In SC, the uncalibrated model simulated corn yield response to N well in 10 years, moderately well in 3 years, and poorly in 2 years; while after calibration the model simulated yields well in 11 years and poorly in 4 years (**Figure 2**). In general the calibrated model captured the trends in the observed variability in corn yields across years (**Supplementary Figure S4A**) as well as the annual yield response to N rates (**Figures 1** and **2**).

#### Simulation of Soybean Yields

Given that the simulation setup was sequential and soybean was part of the CS rotation, the ability of APSIM in simulating soybean yields was also tested. The model simulated no response to N rate applied to the previous corn crop, which agrees with the observed data (**Figure 4**; for individual years see **Supplementary Figure S3**). The agreement in simulated soybean yields was moderate before and after calibration (calibrated RRMSE = 19%; **Figure 3**).

### Simulation of Optimum N Rate and Methods Comparison

#### Site Mean Optimum N Rate

The calibration process improved the prediction of the site mean EONR in the SC but not in CC (**Table 1**; **Figure 3**). The simulated EONR (both calibrated and uncalibrated versions) was overestimated in CC and underestimated in SC (**Table 1**). The absolute difference in site mean EONR between simulated and observed values was smaller in SC; -39 and 18 kg N ha^-1^ for CC and SC, respectively, before calibration and -41 and 10 kg N ha^-1^ for CC and SC, respectively, after calibration (**Table 1**). In addition, the simulated EONR SD was high with the APSIM-Unc, largely due to mis-estimation of some years as non-N responsive.

**Table 1:** Mean economic optimum N rate (EONR, kg N ha^-1^) across 16-years for: observed, Obs; un-calibrated Agricultural Production Systems sIMulator (APSIM) model, Unc; calibrated model, Cal; and the return to N approach from the calibrated model, RTN.

		Obs	Unc	Cal	RTN	Obs-Unc	Obs-Cal	Obs-RTN
			
	Rotation	Mean values					Differences	
Average of years ± SD^1^	CC	188 ± 42	190 ± 82	225 ± 33	176 ± 21	-2	-37	12
	SC	149 ± 48	99 ± 71	137 ± 43	118 ± 30	50	12	31
Pooled^2^	CC	187	226	228	195	-39	-41	-8
	SC	158	140	147	140	18	10	18


*Absolute differences between EONR-Obs and simulations are also shown. Continuous corn, CC and soybean-corn rotation, SC.*

*^1^ Individual annual optimum N rate estimates were averaged across 16-years and the standard deviation (SD) calculated.*

*^2^ Individual annual corn yield values were first averaged across years and then the optimum N rates estimated.*

#### Annual Optimum N Rate

The calculated EONR-Obs (from observations) was highly variable from year to year and ranged from 123 to 268 kg N ha^-1^ in CC and from 42 to 241 kg N ha^-1^ in SC (**Figure 4**). The inter-annual variability in EONR-Obs was greater in SC than in CC (CV of 32 vs. 22%, respectively).

The calculated EONR from the APSIM model followed some of the observed annual trends (**Figure 4**), with the prediction error to be larger in SC than CC (**Supplementary Figure S7**). In CC, the RMSE ranged from 63 kg N ha^-1^ before calibration to 56 kg N ha^-1^ after calibration. In SC, the RMSE ranged from 83 kg N ha^-1^ before calibration to 68 kg N ha^-1^ after calibration. Interestingly, the two methods of calculating EONR from modeled yields (via regression Eqs. 1–3 or via the RTN approach) had similar RMSE and RRMSE values across years, but the annual predictions of optimum N using the RTN approach were less variable across years (**Figure 4**). These results show that for year-to-year simulation of EONR, the calibrated version should be used either via Eqs. 1–3 with regression analysis or the RTN approach. Overall the calibration process reduced the RRMSE in annual EONR predictions by 14.2% in CC and 10.3% in SC (**Supplementary Figure S8**).

The calculated yearly YEONR-Obs (from observation) was less variable compared to the EONR variability (CV of 17 and 12% for CC and SC, respectively, **Figure 4**). The simulated YEONR followed the observed annual trends well (**Figure 4**; RMSE of 1.88 Mg ha^-1^ before calibration and 1.41 Mg ha^-1^ after calibration). The model simulated YEONR was more accurate than EONR. In relative terms, the error in YEONR prediction was about four times lower than the error in EONR prediction (**Supplementary Figures S6** and **S7**). However, there was no correlation between these errors (**Supplementary Figure S6**).

Use of the RTN approach to compute the optimum N rate, and compared to the simulated calibrated values (**Table 1**), produced a closer EONR in CC to the observed EONR (-8 kg N ha^-1^), but a greater difference in SC (18 kg N ha^-1^). Unlike the APSIM-Cal and APSIM-Unc simulations, the RTN-APSIM did not over-estimate EONR in CC, but underestimated in SC (**Table 1**).

### Factors Causing Yearly Variability in Optimal Nitrogen Rate

**FIGURE 5:**
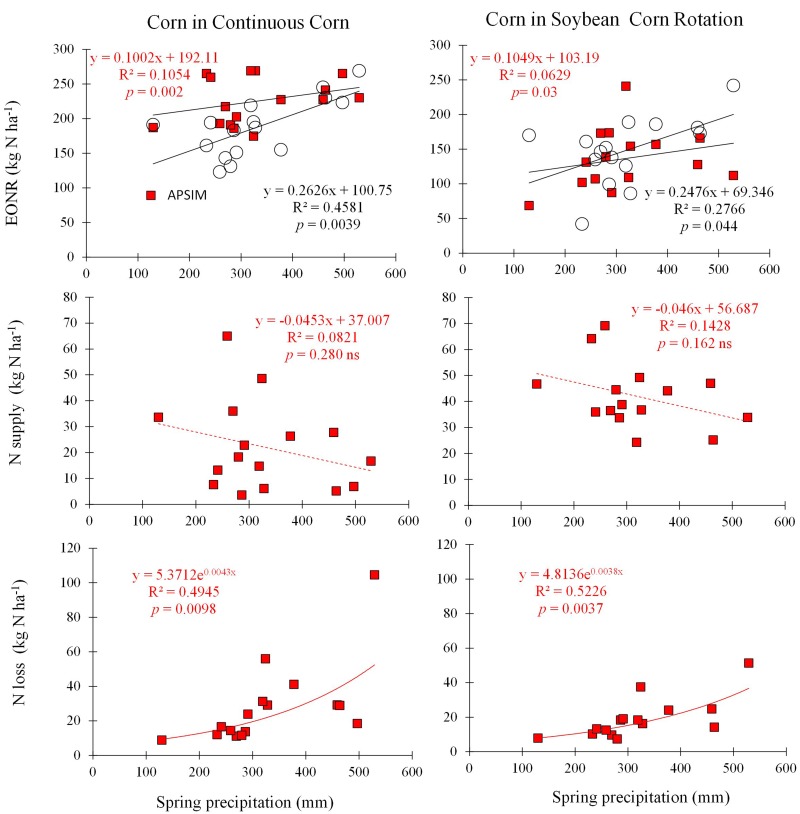
**Cumulative spring precipitation from April 1st to June 30th (every year) versus economic optimum N rate (observed and simulated EONR, circles and squares, respectively; top panels), simulated spring soil N supply (from soil organic carbon mineralization; middle panels), and simulated spring N loss (denitrification and leaching; lower panels) for CC and SC crop sequences**.

The YEONR-Obs (**Supplementary Figure S5**), precipitation (**Figure 5**), and the time of N application (**Supplementary Figure S8**) were explored as possible factors to explain inter-annual variability in EONR. There was a significant positive relationship between spring precipitation and EONR-Obs but the relationship had low predictive power (*p* < 0.05; *R*^2^ = 0.27–0.45; **Figure 5**). Spring precipitation, defined here as precipitation accumulated from April 1 to June 31, was selected from among many other precipitation intervals explored in this study as the best predictor of inter-annual EONR variability (**Supplementary Figure S8**). The YEONR, time of N rate application, the July precipitation (15 days window around corn silking), and combinations of those factors (including spring precipitation) via multi-factor regression modeling did not result in any significant correlation.

The calibrated APSIM version showed a similar relationship between EONR and spring precipitation as with EONR-Obs (**Figure 5**) and therefore the model was used to provide insights into factors causing this relationship. Soil net N mineralization (simulated N supply), and the sum of denitrification and N leaching below 1 m depth (simulated N loss) were used as explanatory variables. The model indicated that the relationship between EONR and spring precipitation was primarily caused by an exponential increase in simulated N loss and to some extent by a reduction in simulated N supply with increasing spring precipitation (**Figure 5**). The model also showed that the rate of the simulated N supply reduction with increased precipitation was similar between rotations. Furthermore model analysis showed that the level of simulated N supply was 50% higher in SC than CC, which explains the lower EONR values typically found in SC systems (**Table 1**; **Figure 3**).

## Discussion

### Calibration Strategy and Steps

Evaluating a model against long-term data is critical when the model is to be used for N management. This is because processes such as N mineralization, require several years to be sufficiently evaluated (Jenkinson et al., 1994; Leigh et al., 1994; Körschens, 2006) and can differentially affect N response among years. Our study is among a few in the literature that tests a process-based model in the long-term (Ma et al., 2007). The long-term data were powerful in detecting weakness in the model, but did not provide guidance on which of the model’s processes or parameters needed to be improved (Kersebaum et al., 2015). Therefore, during calibration we aimed to improve the overall representation of the system based on previous knowledge of the site (for example, C:N ratio of soybean and corn residue, phenology, etc.) rather than just optimizing cultivar parameters by year to better fit the observed data within the study range. This strategy is robust and allows the calibrated model to be used outside the study period (future years) with confidence at this site.

During calibration we implemented the alternate soil water (SWIM) and temperature (soiltemp) models available in the framework, and changed parameters influencing soybean residue C:N ratio (**Table 2**; **Figure 3**). Among changes made in the model, the activation of fluctuating water table via the SWIM soil water model was found to be the most important (e.g., see improvements in yield prediction from 2012 drought in **Figures 2** and **3**). Yet, few models have this capability, despite the great importance of water table depth on water balance, N dynamics, and crop growth (Kalita and Kanwar, 1992; Varvel et al., 1997; Hefting et al., 2004; Kahlown et al., 2005; Nosetto et al., 2009; Portela et al., 2009). Simulations of the groundwater table depth (**Supplementary Figure S2I**) were reasonable judging measurements in nearby sites (Hatfield et al., 1999; Helmers et al., 2012; Archontoulis et al., 2016).

**Table 2:** Statistical evaluation of the uncalibrated and calibrated APSIM model performance in simulating corn and soybean yields by N rate across 16-years.

	Uncalibrated					Calibrated/Validated				
		
	Corn			Soybean		Corn			Soybean	
				
N rate	CC	SC	SC_val^1^	SC	SC_val	CC	SC	SC_val^1^	SC	SC_val
**RMSE (kg ha^-1^)**										
0	1412	946	1365	631	1337	1283	920	1829	560	789
67	1255	806	1996	689	1275	1284	718	1892	663	619
134	1627	1324	2269	672	1264	1085	1466	1259	662	599
201	1870	1586	2528	726	1320	1084	1365	1367	783	716
268	1806	1611	2353	723	1288	946	1387	1452	884	608
Mean	1611	1287	2019	689	1297	1136	1171	1560	722	666
**RRMSE (%)**										
0	39.4	14.7	20.0	21.5	43.5	30.8	12.2	22.9	16.6	22.2
67	17.8	8.8	20.7	23.4	41.1	15.7	6.6	16.9	19.5	17.3
134	18.0	13.3	20.8	22.9	40.9	10.2	15.6	9.9	19.5	16.8
201	19.6	15.2	22.4	25	41.1	9.7	11.2	10.4	23.4	19.3
268	18.2	15.4	20.6	24.8	39.7	8.1	12.3	10.9	26.4	16.3
Mean	20.6	14.4	20.3	23.5	41.3	14.9	11.6	14.2	21.1	18.3


*^1^Dataset used for model validation.*

*Root mean square error, RRMSE; relative root mean square error, RRMSE; continuous corn, CC; corn and soybean phases over the corn-soybean rotation (SC), part of the SC phase-years used for validation (SC_val).*

The simulated soybean residue C:N ratio was initially low (∼20, **Supplementary Figure S2**) compared to literature values (25–40; Al-Kaisi et al., 2005; Bichel, 2013; Li et al., 2013). The low C:N ratio occurred mainly because APSIM supplies enough N through fixation to ensure non-N limiting soybean growth, and thus no response of soybean yield to prior-year N application to corn (potential residual inorganic-N (**Figure 4**; **Supplementary Figure S2**). This effect resulted in simulated luxurious N uptake in plant tissues and therefore low C:N ratio of the soybean residue. After calibration, the soybean residue C:N ratio increased to reasonable values (around 30; **Supplementary Figure S2D**), the simulation of the annual N fixation decreased to realistic estimates (around 180 kg N ha^-1^ year^-1^, Salvagiotti et al., 2008; Christianson et al., 2012), while the model maintained good performance in terms of N fixation and yield response to prior-year corn N fertilization (**Figure 4**; **Supplementary Figures S2G** and **S3**). We believe these changes improve the representation of N fixation (Chen et al., 2016) and soybean residue in the model. However, more experimental work is needed to verify these changes and improve the simulation of soybean rotation effects in APSIM.

### APSIM Performance in Simulating Yields before and after Calibration

This study quantified accuracy of both calibrated and uncalibrated versions in order to show the degree of improvement possible with the use of long-term data. The ability of a model to predict crop yield in any environment depends on the given inputs (soil, weather, and management), how well the model structure represents reality, and the model parameters. To evaluate and test APSIM model performance, we used a system approach that explicitly considered available experimental data (corn and soybean yield, and SOC measurements) and also using literature information to evaluate additional model processes such as N fixation, root/shoot ratio, N concentrations, phenology, and others (**Supplementary Figures S3** and **S4**). Interestingly, the model simulated long-term SOC change equally before and after calibration (RRMSE < 5%). The reason is likely that for modeling SOC, the cumulative carbon input change over time is most important (e.g., Luo et al., 2011). Annual over- and under-prediction of yields and corresponding carbon input are compensated over time if the long-term site mean yield prediction is similar before and after calibration.

The fact that APSIM simulated well yields and crop yield response to N (**Figures 1–3**), provided strong evidence that important soil and crop processes were being accounted for in the model. Otherwise, the model would consistently produce large under- or over-estimated yields values, resulting in different patterns across time compared to the observations (**Supplementary Figure S4A**). It is important to note the good simulation of grain yield with no N fertilizer input across the 16-years (**Supplementary Figure S4A**), which provides evidence that soil N supply and N uptake were well simulated by the model. Furthermore, the model simulated greater net soil N mineralization in the SC rotation than in CC, which is in line with literature reports (Bundy et al., 1993; Schoessow et al., 2010). Difference in net soil N mineralization (caused by residue amount and C:N ratio) was the main cause of EONR difference between CC and SC as the simulated N loss was found to be about the same in both rotations (**Figure 5**).

As expected, APSIM performance in simulating crop yields improved after calibration: RRMSE decreased from 15–30% to below 15%; **Figure 3**; **Table 2**). These performance evaluation results for crop yields are comparable to those reported in the literature for other models (Ahmed et al., 2007; Thorp et al., 2008; Liu et al., 2011; Yang et al., 2014). For a fair judgment of model performance, we should also mention the following assumptions, and those unknowns that may have an impact on model results: (a) we used a fixed cultivar focused on representing well the phenology across the 16-years (**Supplementary Figure S2**); however, different cultivars were used in the experiment (**Supplementary Table S1**) and some probably had different physiological characteristics; (b) there were unknowns in plant population at harvest, tillage date, and depth; (c) there was likely to be abiotic stresses in some years, hail storm damage (2013, **Figure 2**), and lodging issues (2002 and 2004 in CC, **Figure 1**) that were not considered within the model.

Corn yield predictability with calibrated APSIM (in particular for CC) increased at high N rates in particular for CC (see RMSE, **Table 2**). This occurred mainly because at high N rates the model has to account for only water limitations to crop growth, while at low N rates both water and N (and their interactions) become limiting factors to crop growth. This is also in accordance with published results from other crop models (Timsina and Humphreys, 2006; Liu et al., 2011; Yang et al., 2013; Li et al., 2015a,b).

### Modeling Optimal Nitrogen Rate

Simulating EONR was more sensitive and complex, and had more associated uncertainty, than simulating yields (**Supplementary Figures S6** and **S7**). This occurred because identification of the optimum N rate and associated yield in the yield-N response relationship – were quite dependent on the small incremental change (slope) in yield as N rate approached the maximum response. Over- or under-estimation of simulated yields around the optimum N rate resulted in deviations in model-derived EONR values (Eqs. 1–3). For example, in year 2002 the RRMSE for CC yield predictions by the calibrated model across N rates was 8.6% (**Figure 1**). This variation resulted in a 50% RRMSE in EONR prediction and in a 9.3% RRMSE in YEONR prediction. The difficulty in accurately predicting EONR from five simulated yield points is no different than uncertainties associated with the selection of regression equations to describe yield response to N with observed yields, and thus can affect APSIM estimation of the agronomic and economic optimum N rate (Waugh et al., 1973; Cerrato and Blackmer, 1990; Kyveryga, 2005; Scharf et al., 2005; Hernandez and Mulla, 2008).

However, the RTN-APSIM technique that did not use regression fitted equations had similar RMSE and RRMSE values as with EONR-APSIM-Cal, and was in close agreement with the across years mean (pooled) EONR-Obs (**Table 1**). A main difference between the two techniques was that the RTN-APSIM was less variable from year to year, especially for CC (**Figure 4**). The lower inter-annual variability in optimum N estimates from the RTN-APSIM method could be attributed to the N rate increments used in the calculations (5 kg vs. 67 kg N increments) and computation method differences, which can affect the identification of the optimum N point in yield response to N rate (Bachmaier, 2012). We concluded that the differences between simulated and observed annual optimum N rate values (**Supplementary Figure S7**) are due to over- and under-estimates of corn yields, especially surrounding the N rate inflection point, and to a smaller extent, due to the sensitivity of Eqs. 1–3 used to estimate EONR.

The 16-year mean differences in EONRs, especially for RTN-APSIM (**Table 1**), which could be called estimated errors, are acceptable within historical and current N rate ranges (46–56 kg N ha^-1^; ± 23–28 kg N ha^-1^) suggested for corn (Voss and Shrader, 1979; Sawyer et al., 2006) that includes uncertainty in estimation of optimal N (note that the range also depends on the fertilizer: corn price ratio). This means that the APSIM model can be used as a tool to assist optimum N rate recommendations in this USA region.

An important question is how the model can be used to add value within existing N rate guidelines. The main problem with EONR estimation is that the determination is made after crop harvest when yields are known. However, rate guidance is needed before N application in the fall or spring before and after planting. Thus farmers and crop advisers use guidelines based on extensive numbers of N rate research trials (MRTN; Sawyer et al., 2006). This makes the estimation of the site mean EONR very important in this study, given also that a large portion of Midwestern farmers apply N before crop planting. The APSIM model can assist N rate decisions via the following pathways. First, if the objective of long-term experiments is to derive site mean EONR recommendations, then the model can assist in this task (**Figure 3**). Given that the calibration processes improved more the yearly EONR prediction (14.2 and 10.3% reduction in the RRMSE for CC and SC, respectively, see **Supplementary Figure S7**) than site mean EONR prediction (no improvement for CC and 5% reduction in the RRMSE for SC rotation using the pooled mean), this study provides an encouraging result for model usability if only minimum site-information is available.

Second, APSIM has the potential to predict in real-time soil nitrate dynamics within the soil profile and this information could be used to adjust early-to-mid season N application rates (Archontoulis et al., 2016). This approach is currently being used by commercial companies. Third, since APSIM can predict grain yields early in the season using a range of possible weather conditions (actual, historical, future; Archontoulis et al., 2016), it could also predict needed N rates based on yield predictions as it is currently being applied in Australia as decision-support tool (Yield Prophet; Carberry et al., 2009). Nitrogen rate accuracy from yield prediction would be highly dependent on model yield predictability, and needs to be confirmed with an additional study. The value added by models and the accuracy in predicting needed N rates will always be greater when models are supported by local experimental data to periodically check performance and allow updates in the model algorithms or parameters to deal with new genetics and changes in soil and weather over time (Ahuja et al., 2014).

### Causes of Optimal Nitrogen Rate Variability

In addition to predictability, deeper understanding of the factors causing EONR inter-annual variability is important for optimizing agronomic, economic, and environmental outcomes. Among three factors explored with data available for this study (time of N application, optimum yield, and precipitation periods), the cumulative May to June (spring) precipitation explained yearly EONR variability (**Figure 5**). However, the relatively low predictability of this relationship might be due to other confounding factors such as time of N application and planting date, which were not constant over the 16-year period in this study. The relationship between spring precipitation and EONR found in this study agreed with other studies conducted in rainfed environments (Vanotti and Bundy, 1994; Piekielek et al., 1995; Kachanoski et al., 1996; Bundy, 2000; Lory and Scharf, 2003; Sawyer et al., 2006; Scharf et al., 2006), but not with studies conducted in irrigated regions where yield level (optimum yield) was the main driver for the inter-annual variability in EONR (Dobermann et al., 2003; Gehl et al., 2005). Interestingly, the July precipitation which reflects the ± 15 day period around corn silking (see APSIM diagnostics **Supplementary Figure S2**) was not correlated with EONR yearly variability (*R*^2^< 0.25, *p* < 0.05; **Supplementary Figure S9**) despite the great importance of this period for kernel number determination and corn grain yields (Edmeades et al., 2000; Andrade et al., 2002; Calviño et al., 2003). This would be attributed to high soil moisture capacity of the soil and shallow groundwater tables in this region that can compensate for period of water stress and also due to the fact that corn takes up about 70% of its total N uptake by silking (Ciampitti and Vyn, 2012; Woli et al., 2016).

Variability in EONR and its relationship with spring precipitation in the US Midwest has typically been associated in previous research with an increase in N loss with high spring precipitation but previous studies lacked comprehensive measurements (Meisinger, 1984; Eghball and Varvel, 1997; Kay et al., 2006; Lawlor et al., 2008). The APSIM model analysis explicitly quantified the shape and magnitude of N loss per mm of precipitation and indicated that the shape of the relationship is similar in CC and SC systems (**Figure 5**).

The ability of APSIM and other mechanistic process-based models to simulate and explain the effect of precipitation on simulated N loss and supply, and thus, the impact on N response (**Figure 5**) becomes even more relevant with future climate change scenarios. For the US Midwest several studies have predicted higher frequency of both drought and flood events (Schoof et al., 2010; Kunkel et al., 2013; Dai et al., 2015). In this context, long-term simulations with different weather allow to capture the ranges of yield responses to N rates and choose the optimal rate that more frequently provides the best outcomes in terms of higher yield and lower nitrate leaching (Basso et al., 2016). Furthermore, the APSIM model can generate predictions across different weather scenarios that could be used to inform potential need for changes in future N management decisions.

## Conclusion

Model analysis of a 16-year field-experiment dataset that included crop yields and SOC values with five N fertilizer rates and two crop sequences revealed the following main findings:

(1) The fact that APSIM simulated well crop yields and crop yield response to N rate, provided strong evidence that important soil and crop processes were being accounted for in the model;
(2) Model calibration (implementation of SWIM soil water model with activation of soil water table, use of soil temperature 2 model, and improvements in soybean residue C:N ratio) reduced the simulation error (RRMSE) in crop yield prediction by 9% and the annual EONR prediction by 12%. We also found that SOC prediction was insensitive to calibration when long-term mean crop yield was simulated well;
(3) The optimum N rate was higher for CC than SC and according to the model analysis this is associated with higher SOC net mineralization in the SC rotation.
(4) Simulation of EONR was more sensitive and complex than simulating crop yield. Results suggest that for long-term site mean EONR predictions both versions (calibrated and uncalibrated) can be used, while for accurate year-by-year simulation of EONR the calibrated version should be used. Use of the RTN-APSIM approach (small N rate increment with no regression fit) for optimal rate estimation had similar performance compared to EONR-APSIM-Cal approach (five N rate-points and regression fit). A main difference in optimal N rate estimation between the two techniques was that the RTN-APSIM output was less variable from year to year.
(5) Five potential applications were identified where the model could assist N management: (a) estimation of long-term mean EONR; (b) simulation of N dynamics (soil N available and crop N demand); (c) prediction of optimal N using a range of possible weather conditions; (d) simulation of climate change impact on optimal N need;
(6) The APSIM model can be used to explore and explain factors causing inter-annual variability in EONR. For example, the model showed that in rainfed corn-based systems in Iowa, the higher the spring precipitation (April to June) the higher the EONR because simulated N loss via denitrification and leaching increased exponentially while simulated N supply via mineralization tended to decrease.

Finally, for rainfed corn-based systems in the USA Midwest, a combination of process-based modeling, coupled with existing N rate recommendation methods and field data, may be the best approach to fine tune optimal N rate guidance for corn and to develop future management-based strategies under climate change scenarios for maximizing agronomic, economic, and environmental outcomes.

## Author Contributions

JS and DB collected crop data. HP and MC collected soil data. LP, JS, and SA designed the modeling study. LP and SA performed the model analysis. LP synthesized results and constructed tables, figures for the paper. LP and SA created the first draft and all co-authors (JS, DB, RD, HP, MC, PT, and KM) contributed to the final version of the manuscript.

## Conflict of Interest Statement

The authors declare that the research was conducted in the absence of any commercial or financial relationships that could be construed as a potential conflict of interest.

## Footnotes

1 http://websoilsurvey.nrcs.usda.gov/
