# Supplementary material for: Modeling Long-Term Corn Yield Response to Nitrogen Rate and Crop Rotation
Source: Front Plant Sci. 2016 Nov 11;7:1630. doi: 10.3389/fpls.2016.01630 (PMC5104953; doi:10.3389/fpls.2016.01630)
Supplement: Supplementary file 1 [file Data_Sheet_1.pdf]

Supplementary material

Modeling Long Term Corn Yield Response to Nitrogen Rate and Crop

Laila A. Puntel, John E. Sawyer, Daniel W. Barker, Ranae Dietzel, Hanna Poffenbarger, Michael Castellano, Kenneth J. Moore, Peter Thorburn and Sotirios V. Archontoulis

Front. Plant Sci. 7:1630.doi: 10.3389/fpls.2016.01630

Supplementary table S1

Table S1. Corn and soybean management practices available at the time of simulation: planting date, date of N application, N source, hybrid, relative maturity and seed planting density for corn-corn and corn-soybean rotation from 1999 to 2014.

Year	Corn planting date	Date of N application in corn	N source	Corn relative maturity	Soybean planting date
1999	19-May	3-May	urea	107	19-May
2000	5-May	2-May	urea	107	5-May
2001	9-May	1-May	urea	104	9-May
2002	3-May	26-Apr	urea	105	3-May
2003	29-Apr	22-Apr	urea	108	29-Apr
2004	27-Apr	15-Apr	urea	107	11-May
2005	10-May	3-May	urea	105	10-May
2006	9-May	4-May	urea	106	18-May
2007	13-May	1-May	urea	109	18-May
2008	14-May	6-May	urea	111	20-May
2009	5-May	12-May	32% UAN	105	8-May
2010	29-Apr	5-May	32% UAN	105	6-May
2011	11-May	19-May	32% UAN	106	19-May
2012	10-May	10-May	32% UAN	105	15-May
2013	24-May	13-Jun	32% UAN	104	13-Jun
2014	7-May	6-May	urea	105	20-May

Supplementary table S2

Table S2. APSIM corn and soybean cultivar and crop model specific parameter values used in this study. When more than one value is given (see soybean), this means that there is an array of values for the specific parameter.

Acronym	Value	Unit
Corn		
tt_emerg_to_endjuv (thermal time from emergence to end juvenile)	250	ºC-days
tt_flower_to_maturity (thermal time from flowering to phys maturity)	812	ºC-days
head_grain_no (potential kernel number per ear)	800	#
grain_gth_rate (grain growth rate)	9.17	mg/rain/day
tt_flower_to_start_grain (thermal time from flowering to start grain fill)	170	ºC-days
tt_maturity_to_ripe (thermal time from maturity to harvest)	150	ºC-days
Soybean		
x_pp_hi_incr (photoperiod)	1, 24	Hours
y_hi_incr (daily rate of harvest index)	0.01, 0.01	1/days
x_hi_max_pot_stress (stress index)	0.0, 1.0	(-)
y_hi_max_pot (maximum value for harvest index)	0.5, 0.5	(-)
tt_emergence (thermal time to emergence)	100, 100	ºC-days
x_pp (photoperiod levels)	13.59, 14.6, 15.6, 16.6	Hour
y_tt_end_of_juvenile[1] (thermal time to juvenile)	100, 133, 200, 400	ºC-days
y_tt_floral_inititation (thermal time from end of juv to floral initiation)	128, 171, 256, 512	ºC-days
y_tt_flowering (thermal time from flowering to start grain fill)	246, 328, 492, 1312	ºC-days
y_tt_start_grain_fill (thermal time from start to end of grain fill)	499, 666, 999, 2664	ºC-days
tt_end_grain_fill (thermal time from end grain fill to maturity)	20	ºC-days
tt_maturity (thermal time from maturity to harvest)	70	ºC-days
node_sen_rate (node senescence rate)	95	ºC-days node^{-1}
Twilight (twilight)	0	(-)
x_stage for N fixation (crop stage number)	3, 4, 5, 6, 7	stage #
N_fix_rate (Nn fixation rate)	0.0006, 0.0016, 0.0016, 0.0009	gN/gDM
x_stage for N concentration (crop stage number)	3, 6, 9	stage #
y_n_conc_min_leaf (minimum N concentration in leaves)	0.02, 0.01, 0.0085	gN/gDM
y_n_conc_crit_leaf (critical N concentration in leaves)	0.06, 0.05, 0.02	gN/gDM
y_n_conc_max_leaf (maximum N concentration in leaves)	0.06, 0.05, 0.025	gN/gDM
y_n_conc_crit_stem (critical N concentration in stems)	0.03, 0.02, 0.008	gN/gDM
y_n_conc_max_stem (minimum N concentration in stems)	0.03, 0.02, 0.008	gN/gDM
y_n_conc_crit_pod (critical N concentration in pods)	0.06, 0.06, 0.005	gN/gDM
y_n_conc_max_pod (maximum N concentration in pod)	0.06, 0.06, 0.008	gN/gDM

Supplementary table S3

Table S3. Soil profile values from the initialization period (1993 to 1999). The values refer to the start of the simulation on 1/1/1999. BD, bulk density; LL, lower limit; DUL, drained upper limit; SAT, saturated volumetric water content; SW, soil water ; Corn and Soy KL, parameters defining capacity to extract water per day; OC, soil organic carbon; Finert, inert of soil organic C (not decomposing); Fbiom, microbial SOC (fast decomposing); Hum, humic SOC (medium decomposing); and NO_3-N, soil nitrate.

Soil layer	BD	LL	DUL	SAT	SW	Corn KL	Soy KL	OC	Finert	Fbiom	Hum	NO_3-N
cm	Mg m^{-3}	---------------- mm mm^{-1}-------------				d^{-1}	d^{-1}	g $100g^{-1}$	-------- kg C ha^{-1} ---------			Kg ha^{-1}
0 to 8	1.300	0.164	0.299	0.459	0.275	0.080	0.080	2.00	8008	1183	11609	0.25
8 to 16	1.300	0.164	0.299	0.459	0.263	0.075	0.075	1.98	8442	741	11345	0.30
16 to 31	1.367	0.159	0.296	0.434	0.276	0.070	0.070	1.66	16851	1004	16054	0.60
31 to 54	1.425	0.145	0.286	0.413	0.283	0.060	0.060	1.39	29835	293	14911	1.00
54 to 74	1.450	0.133	0.278	0.403	0.278	0.050	0.050	1.15	26784	171	6395	1.20
74 to 102	1.550	0.132	0.277	0.365	0.277	0.043	0.043	0.43	15020	169	3631	0.40
102 to 120	1.600	0.132	0.276	0.346	0.276	0.035	0.035	0.15	3417	73	830	0.05
120 to 150	1.600	0.132	0.276	0.346	0.323	0.030	0.000	0.15	6178	64	959	0.05
150 to 199	1.600	0.132	0.276	0.346	0.346	0.000	0.000	0.15	11416	20	324	0.00

Supplementary figure S1

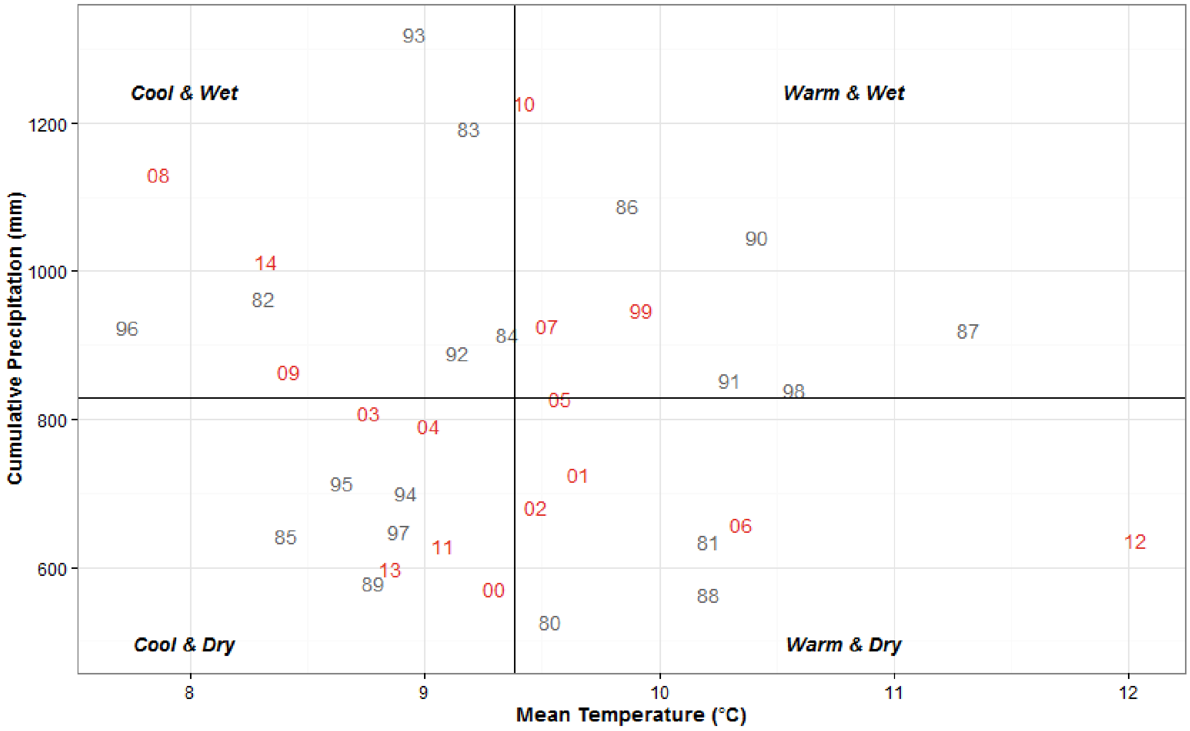

Figure S1. Cumulative annual precipitation and mean temperature in Ames, Iowa, USA. The long term average cumulative precipitation and temperature across years (1980-2014) are shown with the vertical and horizontal lines, respectively. These average values were used as classification criteria to separate years into warm, cool, dry, and wet. Years shown in red represent the years used in this study (1999–2014).

Supplementary figure S2 and APSIM diagnosis

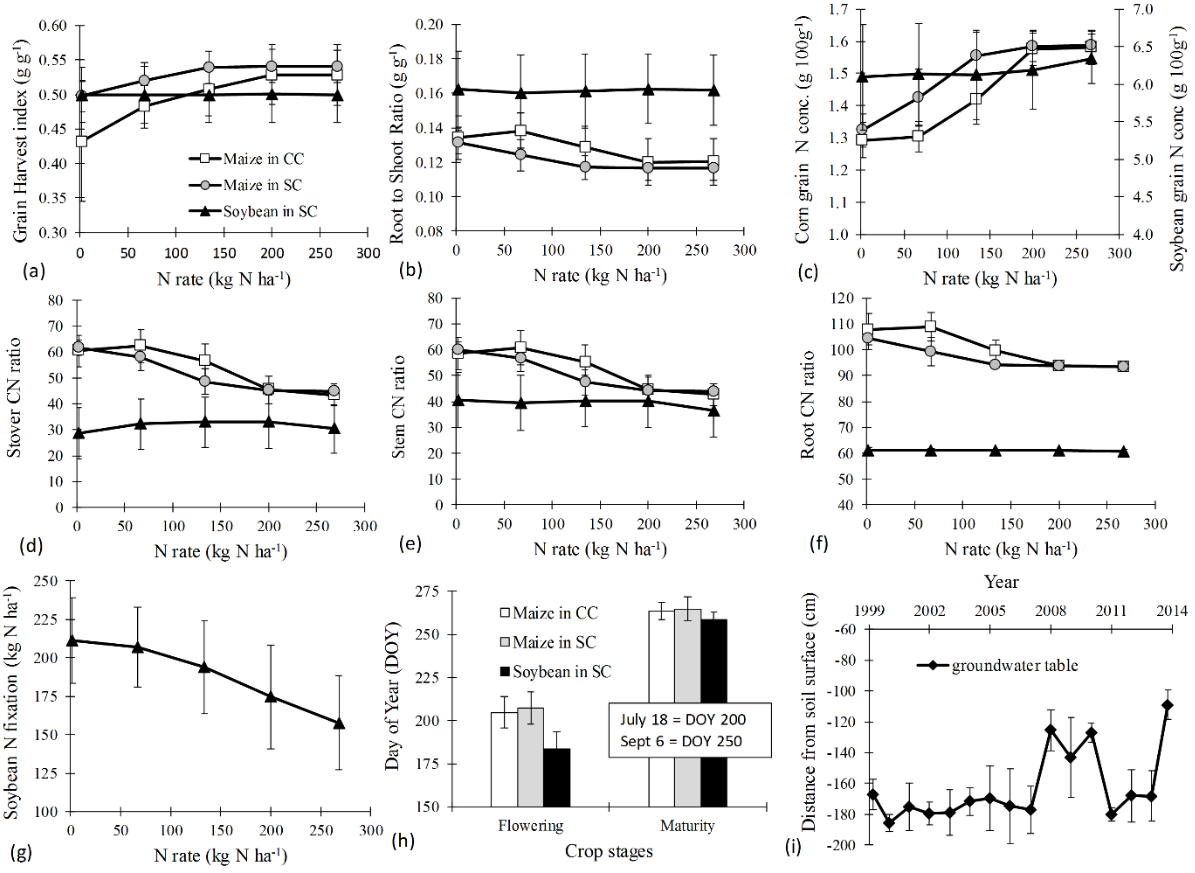

Figure S2. Simulation of grain harvest index at harvest (a), root to shoot ratio at harvest (b), grain N concentration at harvest (c), stover (above ground biomass minus grain), stem and root C to N ratio at harvest (d, e, and f, respectively), soybean N fixation (g), time to flowering and maturity (h) and year to year fluctuation of groundwater table at harvest (i, average trend across cropping systems and N-rates). The points (squares, cycles and triangles) are average values across experimental years (1999-2014) and the corresponding vertical bars represent the standard deviation. CC: continuous corn, SC: soybean-corn rotation, and SC_val: soybean-corn rotation data set used for validation.

APSIM diagnostics

One of the major goals in testing model performance is to evaluate the behavior of the model. Figure S1 illustrates simulated results (1999–2014) from the calibrated model for key model variables. In the absence of specific measurements, these results were judged by experts and literature information and found to be reasonable. The simulated grain harvest index ranged from 0.43 to 0.55 and was affected by N rate in corn. The root to shoot ratio at harvest showed a small decline with N application rate and was about 0.13 and 0.16 for corn and soybean, respectively. The grain N concentration was different between corn and soybean crops (1.5 vs. 6.5%, respectively) and it showed a positive response to N rate. Stover (above ground biomass minus grain), stem, and root N concentration and C:N ratios were different between crops, showed a

response to N-rate while their simulated values were within the range of values reported in the literature (Ciampitti and Vyn, 2012; Al-Kaisi et al., 2005; Salvagiotti et al., 2008). The simulated soybean N fixation was on average 180 kg N/ha and showed a strong response to residual N, in line with literature findings (Salvagiotti eta al., 2008). Simulation of flowering and physiological maturity for both crops reflected very well what is usually observed in this region. Finally the groundwater table varied from year to year and was shallower in wetter years such as 2008, 2010 and 2014 (Fig. S1i, and Fig. 1).

References cited:

Ciampitti, I.A., Vyn, T.J. (2012). Physiological perspectives of changes over time in maize yield dependency on nitrogen uptake and associated nitrogen efficiencies: A review. Field Crops Res. 133, 48-67.

Al-Kaisi, M.M., Yin, X.H., Licht, M.A. (2005). Soil carbon and nitrogen changes as affected by tillage system and crop biomass in a corn–soybean rotation. Appl. Soil Ecol. 30, 174–191.

Salvagiotti, F., Specht, J.E., Cassman, K.G., Walters, D.T., Weiss, A., Dobermann, A. (2008). Growth and nitrogen fixation in high yielding soybean: impact of nitrogen fertilization. Agron J., 101, 958–970.

Supplementary figure S3

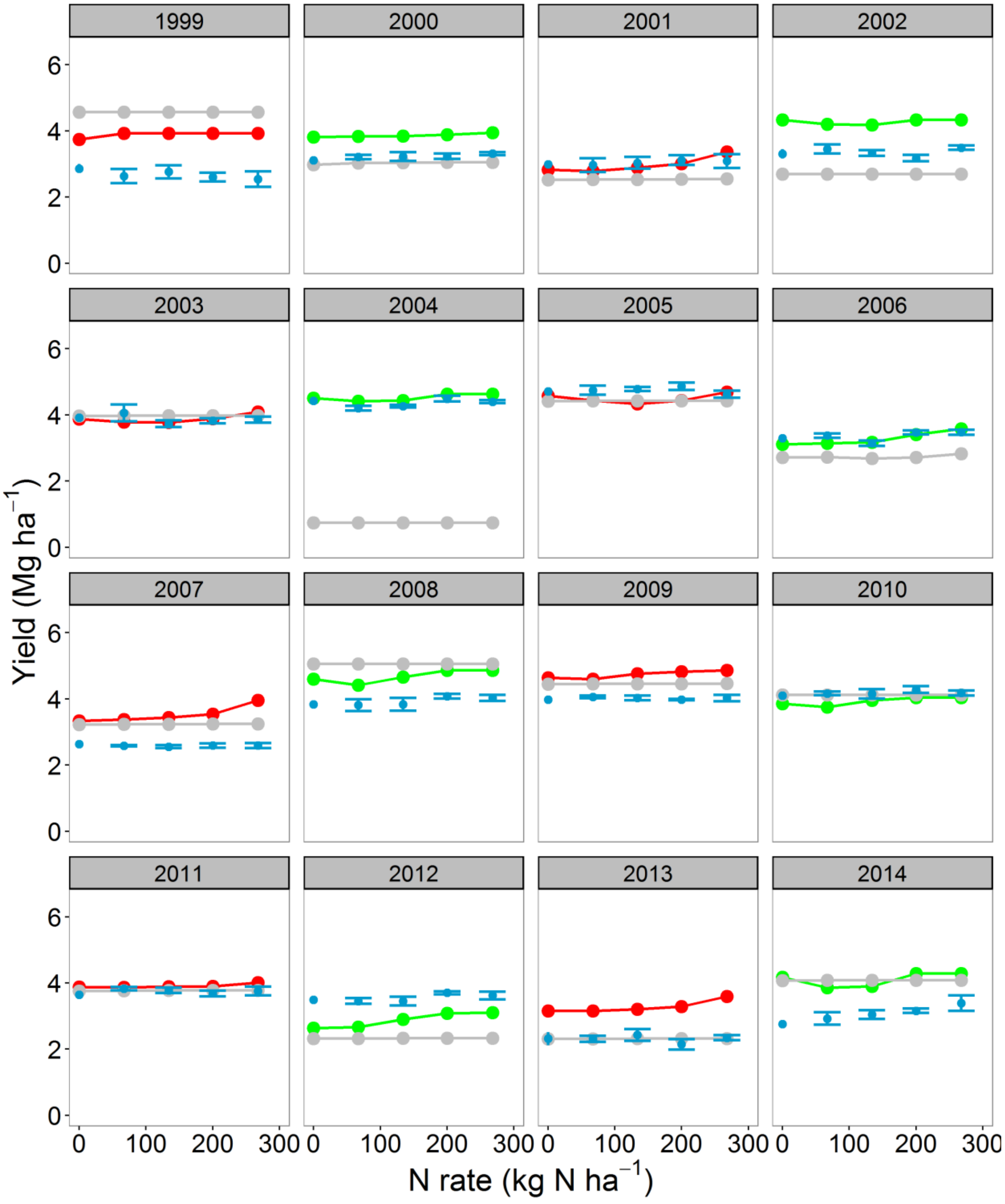

Figure S3. Soybean grain yield versus N rate applied to the previous year corn in the soybean-corn rotation. The blue points with standard errors (n=4) indicate the measurements. The grey, red, and green connected points indicate uncalibrated, calibrated and validated simulations from the APISM model.

Supplementary figure S4

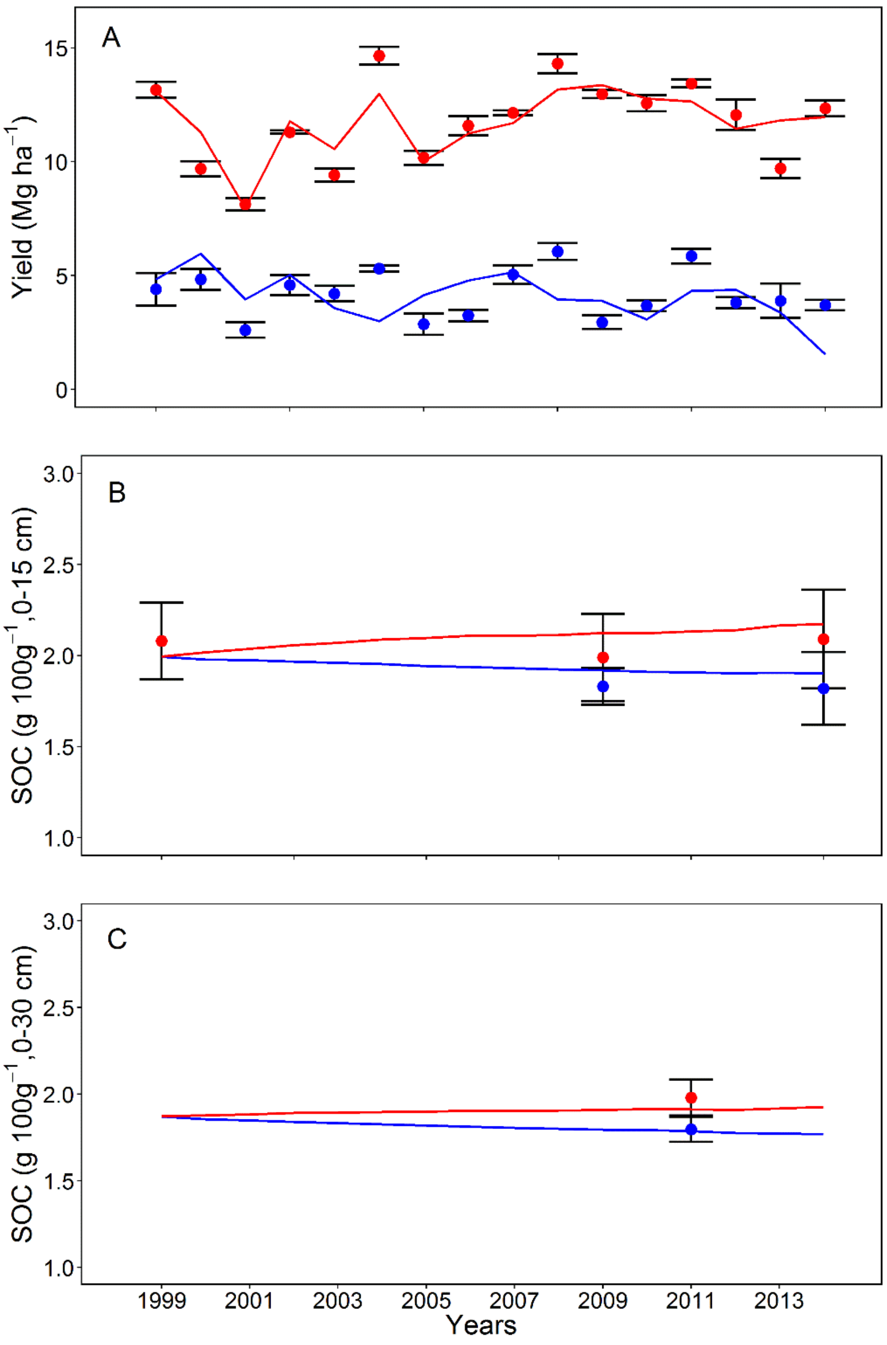

Figure S4. Temporal variability in corn yield (A) and soil organic carbon (SOC) changes at 0-15 cm (B) and at 0-30 cm (C) for the continuous corn system. Continuous lines are simulations from the calibrated APSIM model and points are observations. Color blue and red refers to 0 and 268 kg N ha-1 treatments, respectively. Vertical bars represent the standard error of the observed mean.

Supplementary figure S5 and S6

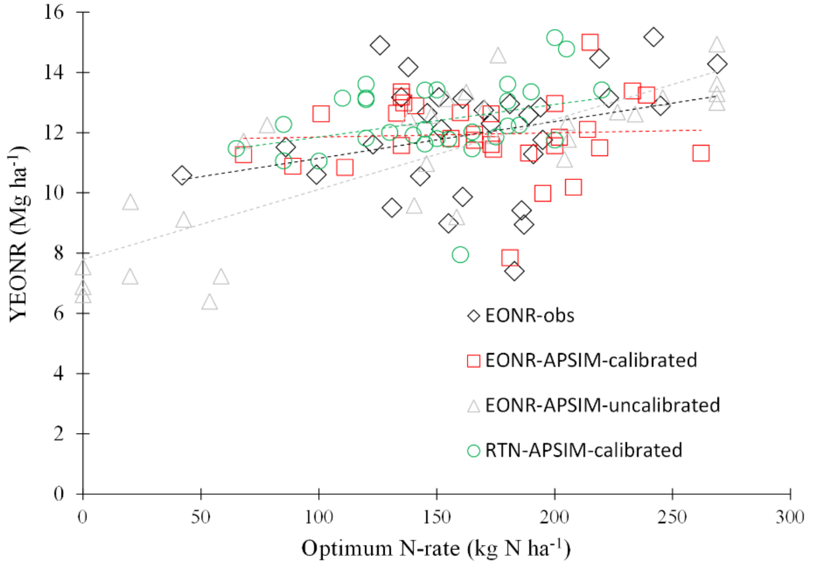

Figure S5. Relationship between yield at the economic optimum N rate (YEONR) and the optimum N-rate for continuous corn and soybean-corn rotation. Dashed lines indicate non-significant trends.

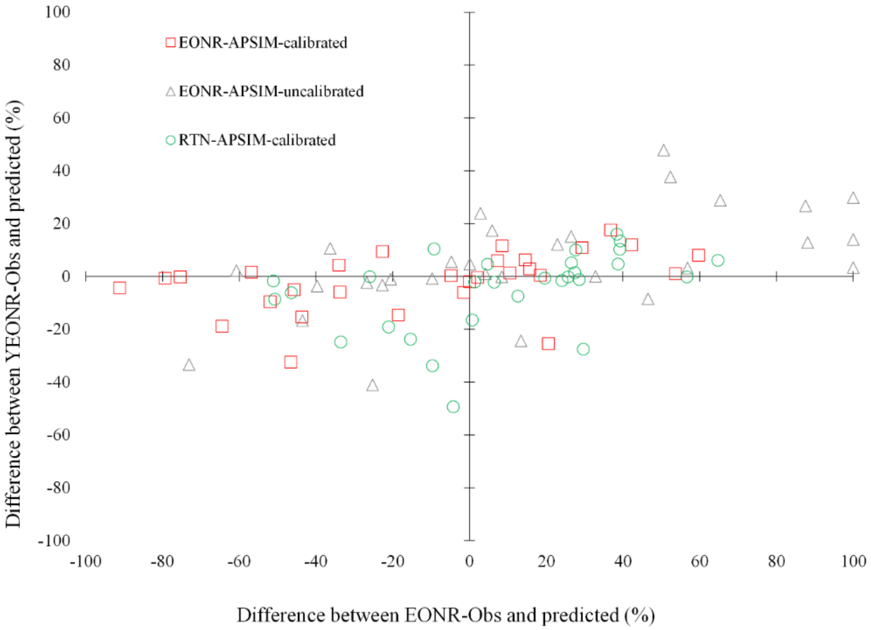

Figure S6. Relative difference between predicted and observed economic optimum N rate (EONR, x-axis) and between simulated and observed yield at optimum N rate (YEONR, y-axis).

Supplementary figure S7

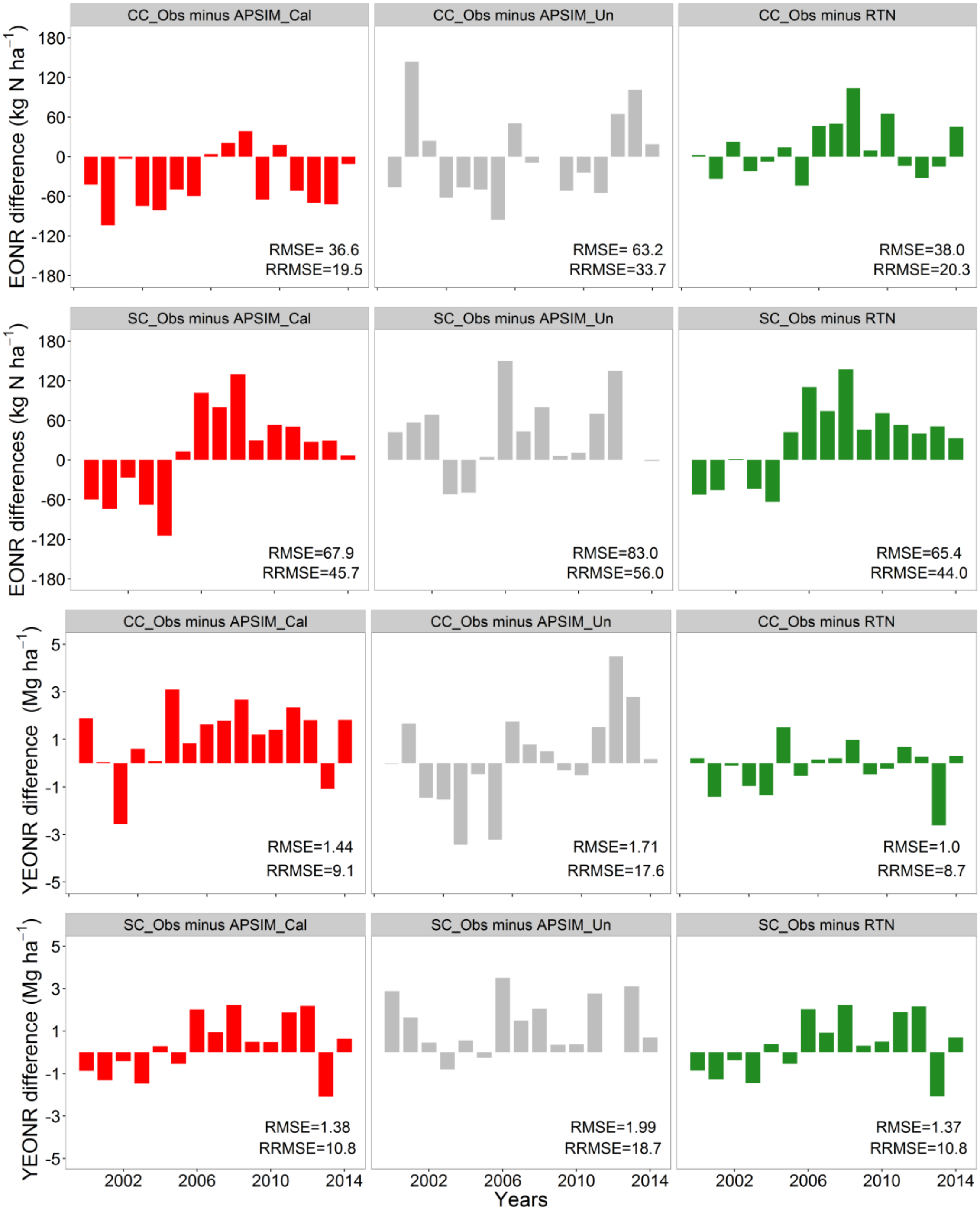

Figure S7. Annual difference between simulated and observed economic optimum N rate (EONR) and yield at the EONR (YEONR). The first part of each acronym within a panel heading refers to the crop sequences (CC, continuous corn and SC, soybean-corn). The second part refers to the differences being shown; for example Obs minus APSIM_cal = EONR-Obs minus EONR-APSIM_cal, and RTN refers to return to N approach from the calibrated model.

Supplementary figure S8

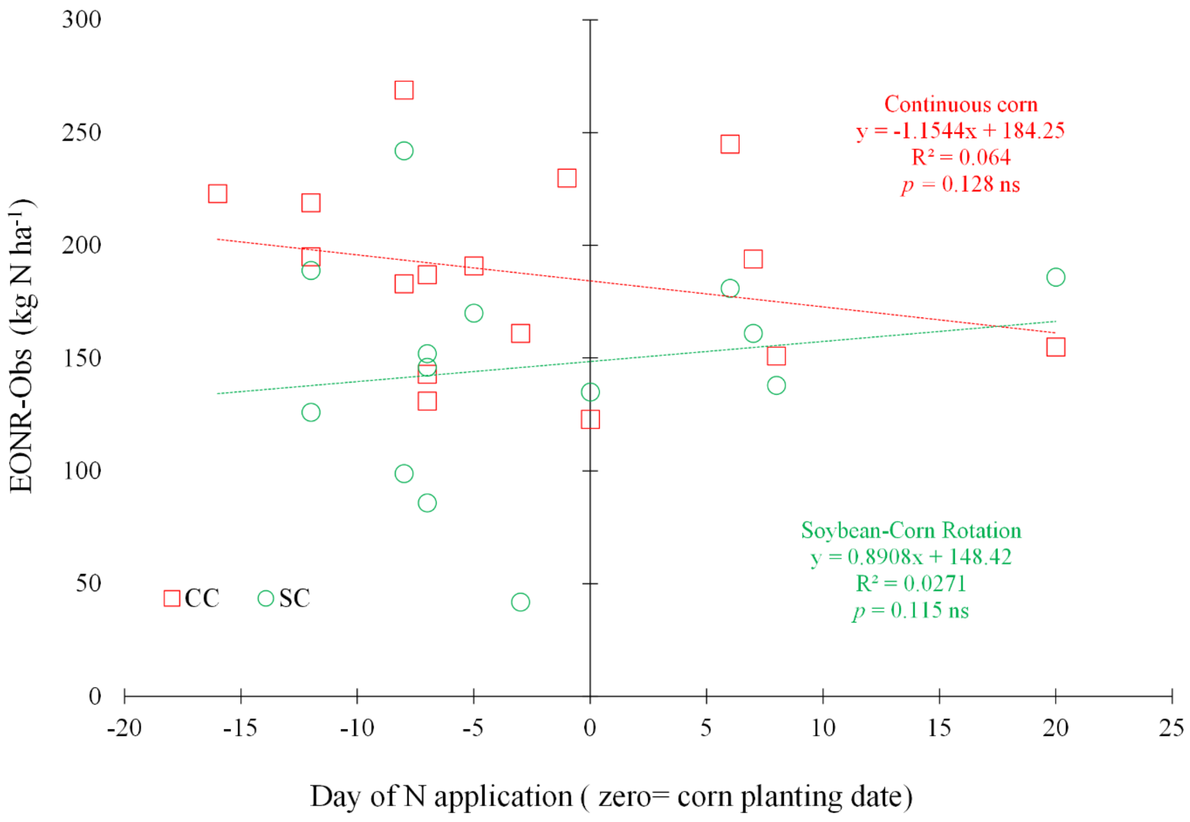

Figure S8.Economic optimum N rate (EONR) from observations versus time of N application relative to corn planting date. Dotted lines indicate non-significant trends.

Supplementary figure S9

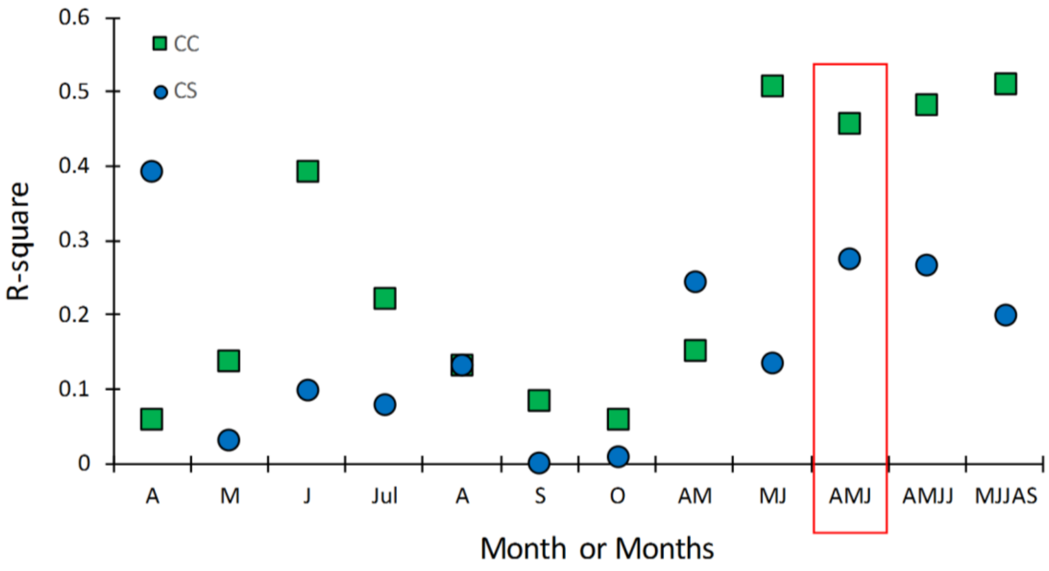

Figure S9. The R-square values derived from linear regression between observed economic optimum N rate (EONR-Obs) and precipitation for different months or a combination of months. A: April, M: May, J: June, Jul: July, A: August, S: September, O: October, AM: April to May, MJ: May to June, AMJ: April to June, AMJJ: April to July and MJJAS: May to September. Red box indicates the selected period used for further analysis (see main text, Fig. 9).
